# Efficacy of One‐stage Paravertebral Approach using a Micro‐Tubular Technique in Treating Thoracic Dumbbell Tumors

**DOI:** 10.1111/os.12991

**Published:** 2021-05-04

**Authors:** Rui Wang, Yan Chen, Zeyan Liang, Weizhong Yang, Chunmei Chen

**Affiliations:** ^1^ Department of Neurosurgery Fujian Medical University Union Hospital Fuzhou China

**Keywords:** Dumbbell tumor, Micro‐tubular technique, Thoracic spinal tumor, Tubular spinal surgery

## Abstract

**Objective:**

The aim of the present study was to investigate the feasibility and efficacy of one‐stage surgical resection of thoracic dumbbell tumors using a paravertebral approach and a micro‐tubular technique.

**Methods:**

Clinical data of thoracic dumbbell tumors resected using a paravertebral approach and a micro‐tubular technique (14 mm, non‐expandable type) in the Department of Neurosurgery at our hospital from July 2014 to July 2019 were retrospectively analyzed. Tumors were found between T1 and T12 vertebrae. Operation time, blood loss, hospitalization, recovery of neurological function, complications, the Japanese Orthopaedic Association (JOA) score, and the visual analogue scale (VAS) score were used to evaluate clinical efficacy.

**Results:**

In all 31 cases, tumors were completely resected in one operation, with a mean blood loss of 53.23 ± 33.08 mL (20–150 mL) and a mean operation time of 95.16 ± 20.31 min (60–180 min). According to the Eden classification, there were four type II cases, 16 type III cases, and 11 type IV cases. The incidence of tumors in the lower thoracic segment (T8–T12) was 51.6% (16/31 cases), while the incidences in the upper thoracic segment (T1–T4) and middle segment (T5–T8) were 25.8% (8/31 cases) and 22.6% (7/31 cases), respectively. Pathological diagnoses were schwannoma (n = 22), gangliocytoma (n = 4), metastatic tumor (n = 2), neurofibroma (n = 1), granuloma (n = 1), and lipoma (n = 1). After surgery, symptoms were relieved in all patients. VAS and JOA scores significantly improved (*P* < 0.001). There was no pleural or lung injury, and there were no complications, such as cerebrospinal fluid leakage. The average follow‐up duration was 29 months (13–59 months), during which time no tumor recurrence or spinal instability occurred. The group of Eden type II tumors had lower JOA scores at 12 months postoperatively, longer operation times, and more estimated blood loss compared with other groups (*P* < 0.05). There were no significant influences on VAS scores at 12 months postoperatively and postoperative hospital stay from the different types of tumors.

**Conclusion:**

The paravertebral approach with a micro‐tubular technique is a safe and effective minimally invasive surgical approach for thoracic dumbbell tumors that allows one‐stage tumor resection using a single incision. Using this approach significantly reduces intraoperative blood loss and postoperative complications, shortens hospital stay, and reduces the rates of postoperative spinal instability.

## Graphical abstract

Preoperative T2‐weighted MRI on axial (A) and coronal (B) planes, revealing a dumbbell tumor in the left T8–9 foramen (arrows). Rebuilding the relationship between the vertebrae (yellow) and the dumbbell tumor (purple) and designing the operational approach with micro‐tubule (number 1, brown and number 2, green) using 3D software. Location of the tumor after partial resection of vertebrae(G).

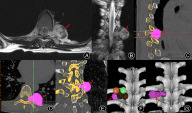

## Introduction

Most thoracic dumbbell tumors are benign; the main symptoms are local pain and weakness, which are mostly caused by compression of the spinal cord and spinal nerves^1^, ^2^. Although the paravertebral parts of dumbbell tumors rarely cause symptoms, their expansive growth in the thoracic cavity often leads to difficulty in surgical resection. Eden classification is the most commonly used classification method for thoracic dumbbell tumors. The Eden classification of spinal dumbbell tumors includes four types: type I, intradural and extradural; type II, intradural and extradural + paravertebral; type III, epidural + paravertebral; and type IV, intervertebral foraminal + paravertebral^3^.

Thoracic dumbbell tumors can compress the spinal cord and nerve roots in the spinal canal and paravertebral tissues, such as the lungs and aorta in the thoracic cavity. This makes surgery risky and difficult; hence, combined or staged surgery is often performed^1^, ^4^, ^5^, ^6^, ^7^. With the development of microsurgery and spinal biomechanics, patients are increasingly being treated with one‐stage surgery. Among the various surgical procedures for the treatment of thoracic dumbbell tumors, the posterior midline approach combined with thoracoscopic surgery has become increasingly common, with most cases undergoing combined surgery jointly performed by neurosurgeons and thoracic surgeons.

We analyzed existing clinical data of patients who had undergone one‐stage surgical resection for thoracic dumbbell tumors through the paravertebral space using a micro‐tubular technique to: (i) discuss the surgical indications and limitations of a micro‐tubular technique for thoracic dumbbell tumor; (ii) describe the technical details of this operation; and (iii) summarize the related effects and common complications of this operation.

## Materials and Methods

### Patient Information

We performed a retrospective review of 31 cases of thoracic dumbbell tumors that were resected using a paravertebral approach and a micro‐tubular technique (14 mm, non‐expandable type) in the Department of Neurosurgery at our hospital from July 2014 to July 2019.

Approval for this study was granted by the Ethics Committee of Fujian Medical University Union Hospital, Fuzhou, China (2020YF023‐01).

#### Inclusion Criteria and Exclusion Criteria

Inclusion criteria were set according to the PICOS (patients, intervention, comparison, outcomes, and study design) principle: (i) intraspinal tumor diameter <2.5 cm and extraspinal tumor diameter <4.0 cm; and (ii) no bone destruction in vertebral bodies in the segment with the tumor.

The exclusion criteria were: (i) invasion of the tumor into the vertebral bodies, with serious bone destruction; (ii) invasion of the tumor into the great vessels of the pleura and thoracic cavity; and (iii) severe thoracic scoliosis and spinal instability.

#### Clinical Data and Imaging Examination

Various clinical manifestations and presentations of the patients were also assessed. Radiographic examination, CT three‐dimensional reconstruction, and MRI with enhanced scanning were performed on the thoracic vertebrae before surgery to obtain a definitive diagnosis.

### Surgical Technique

#### Plan of Paravertebral Approach

**Fig 1:**
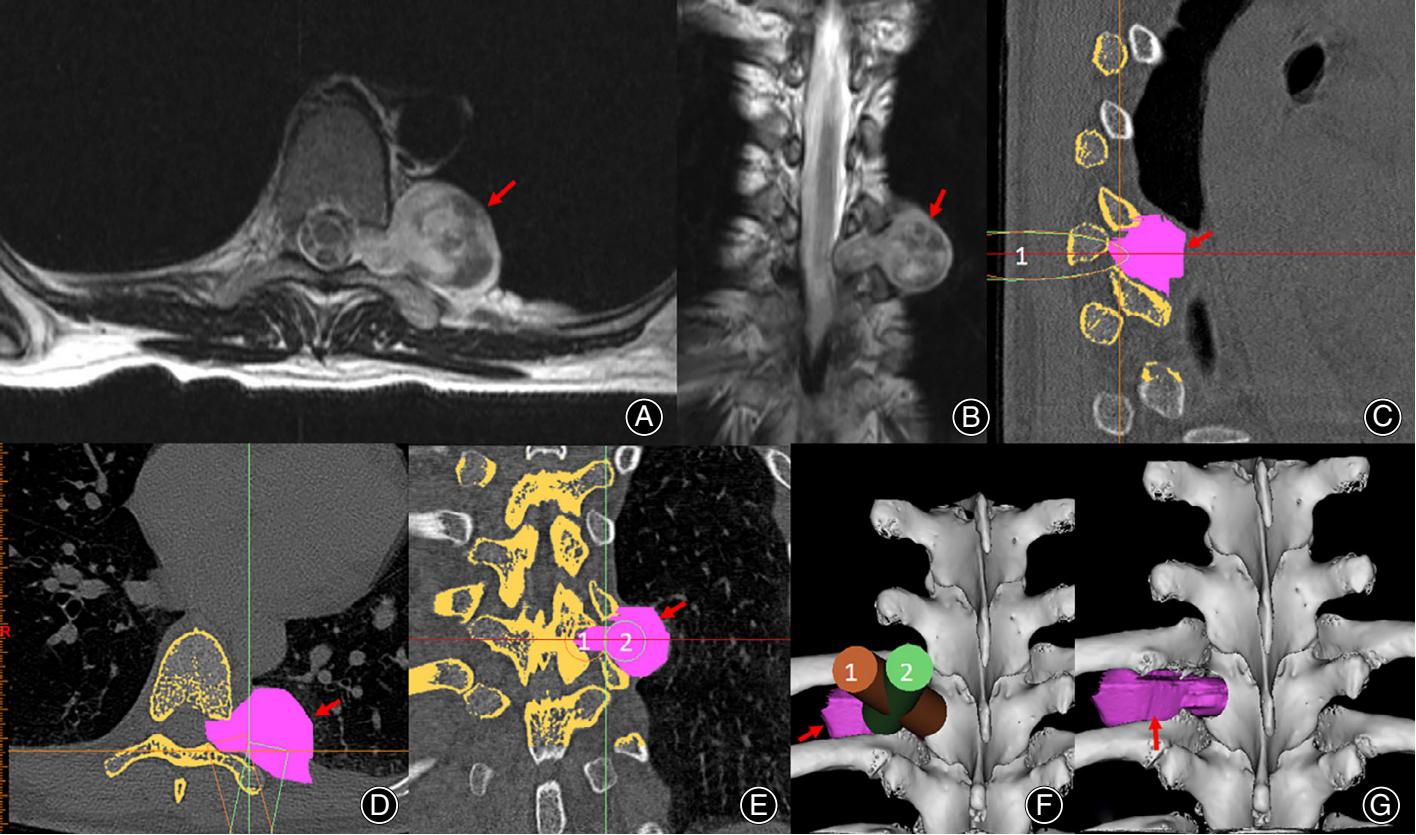
Preoperative T2‐weighted MRI on axial (A) and coronal (B) planes, revealing a dumbbell tumor in the left T8–9 foramen (arrows). Rebuilding the relationship between the vertebrae (yellow) and the dumbbell tumor (purple). Designing the operational approach with micro‐tubule (number 1, brown and number 2, green) using 3D software. Location of the tumor after partial resection of vertebrae (G).

The tumors were resected through the paravertebral approach, using a micro‐tubular technique, which is our self‐developed paravertebral approach tubular system (diameter: 14 mm, non‐expandable). Before surgery, the distance from the skin to the intraspinal and extraspinal tumors and the distance from the side of the tumors to the midline were measured from preoperative imaging data, and the deletesurgical path of the paravertebral approach was designed (Fig. 1).

#### Anesthesia and Position

After induction of general anesthesia, the patients were placed in the prone position to minimize thoracic lordosis and to avoid compressing the abdomen.

#### Approach and Exposure

During surgery, the operating segment was located using plain X‐rays, and a 2.0‐cm straight surgical incision was made 2.0–3.5 cm lateral to the midline (adjusted according to the patient's body habitus). A paravertebral expansion sleeve was used to separate the muscle layer stepwise using blunt dissection, the working tubule was inserted and fixed, and an X‐ray system was again used to locate and confirm the operating segment.

#### Resection of Tumor within the Spinal Canal

**Fig 2:**
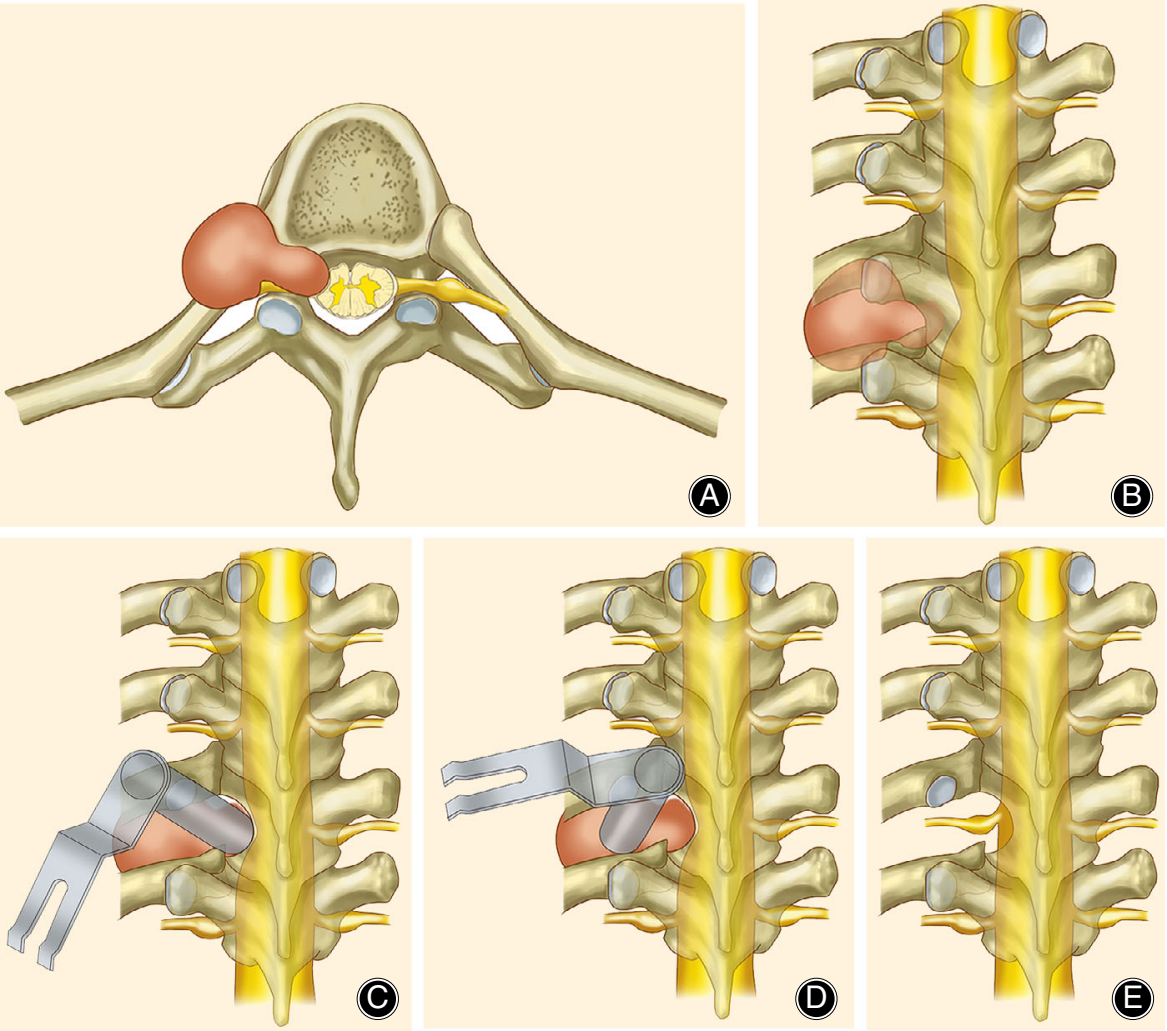
Surgical diagrams illustrating the operation process. (A) and (B) A dumbbell tumor in thoracic vertebrae on axial and coronal planes, revealing a dumbbell tumor in the left thoracic intervertebral foramen. (C) After inserting the micro‐tubule, the direction of the operating tubule under the microscope was adjusted to reach the lamina, part of the bone of the lamina was ground off, and the tumor was excised within the spinal canal or inside the intervertebral foramen. (D) The direction of the micro‐tubule was adjusted to reach the lateral side of the intervertebral foramen; a microdrill was used to grind off part of the bone on the lateral side of the costotransverse joint and the bone on the superior edge of the lower rib; the muscle tissue in the intercostal space was dissected to expose the outer part of tumor and the lateral side of the intervertebral foramen; and the capsule was resected after piecemeal resection of the tumor in the capsule to protect the pleura. (E) The medial and lateral sides of the intervertebral foramen were explored. After confirming that the tumor was completely removed, the operating tubule was withdrawn.

If the tumor was Eden type II or III, the direction of the operating tubule under the microscope was adjusted to reach the lamina, part of the bone of the lamina was ground off, and the tumor was excised within the spinal canal or inside the intervertebral foramen. If the tumor was Eden type II, the dura mater was also opened, the tumor under the dura mater was resected piece by piece, and the dura mater was then tightly sutured. The blood vessels supplying the tumor were traced along the tumor‐bearing nerve and tumor capsule and were ligated (Fig. 2A–C).

#### Resection of Paravertebral Tumor

**Fig 3:**
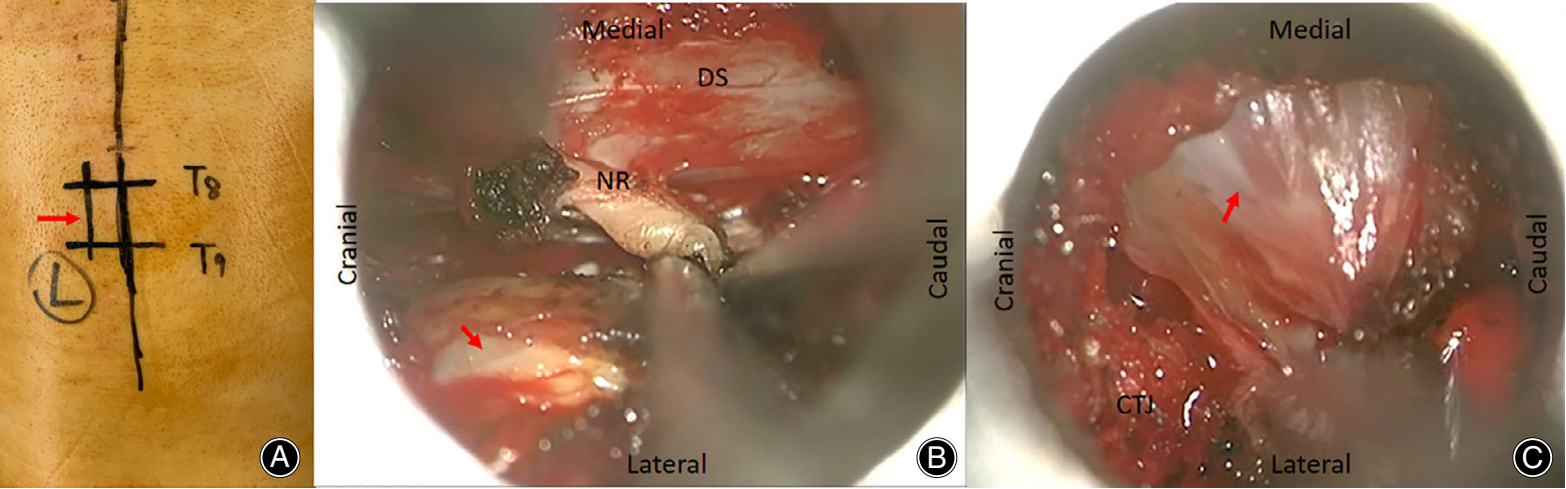
A 2.0‐cm straight surgical incision was made 2.0‐cm lateral to the midline (A). After inserting the micro‐tubule, the relevant anatomy was exposed (B): dural sac (DS), nerve root (NR), and intraspinal tumor (arrow). After changing the angle of the tubule, the costotransverse joint (CTJ) of T8 and the lateral border of the extraspinal tumor (arrow) were exposed (C).

The direction of the micro‐tubule was adjusted to reach the lateral side of the intervertebral foramen; a microdrill was used to grind off part of the bone on the lateral side of the costotransverse joint and the bone on the superior edge of the lower rib; the muscle tissue in the intercostal space was dissected to expose the outer part of the tumor and the lateral side of the intervertebral foramen; and the capsule was resected after piecemeal resection of the tumor in the capsule to protect the pleura. The medial and lateral sides of the intervertebral foramen were explored. After confirming that the tumor was completely removed, the operating tubule was withdrawn, and a paravertebral surgical cavity drainage tube was inserted. The paravertebral muscles were approximated, and the fascia, subcutaneous tissues, and skin were sutured in sequence (Figs 2D,E and 3).

### Postoperative Management

Postoperatively, a small dose of methylprednisolone (40–80 mg) was administered intravenously for 2 days, and the drainage tube was removed after 24 h. Patients were encouraged to exercise their lower limbs in bed and to get out of bed and walk as soon as possible. Patients were also followed up regularly after discharge.

### Outcome Measures

Recovery of neurological function before and after surgery was compared using Japanese Orthopaedic Association (JOA) and Visual Analogue Scale (VAS) scores. The JOA score was focused on lower motor and sensory function. Bladder function was related to the thoracic dumbbell tumor. The VAS was used to assess lower back pain and leg pain. VAS and JOA scores were recorded preoperation and at the final follow up after the operation.

### Statistical Analysis

Data were analyzed using SPSS version 23.0 (IBM, Armonk, NY, USA). Mean values were reported as mean ± standard deviation. Median values were reported with the range. The preoperative and postoperative follow‐up VAS score and JOA score data were compared using a paired *t*‐test. *P*‐values <0.05 indicated a statistically significant difference. An exploratory subgroup analysis is carried out to investigate whether the treatment effect varied over a specific subgroup of patients.

## Results

### Patient Characteristics

Among the 31 patients (average age, 49.9 [range, 21–77] years) included in this study, 12 were men and 19 were women. The average duration of the disease was 1.4 years (range, 1 month–4 years). Clinical manifestations included: chest pain, back pain, and paresthesia (n = 24); neck pain and numbness in both upper limbs (n = 2); and reduced muscle strength in both lower limbs accompanied by sphincter dysfunction (n = 5). According to the Eden classification^3^, there were four type II cases, 16 type III cases, and 11 type IV cases. Tumors were found between T1 and T12 vertebrae (Table 1).

**TABLE 1:** Clinical data of patients

Variable	Measurement (number or mean ± standard deviation)
Gender	
Male	12
Female	19
Age	49.94 ± 14.54 years
Eden type	
Type II	4
Type III	16
Type IV	11
Tumor size (maximum diameter)	3.06 ± 0.66 cm
Pathology	
Schwannoma	22
Gangliocytoma	4
Metastatic tumor	2
Neurofibroma	1
Granuloma	1
Lipoma	1
Tumor site	
T1–T2	4
T2–T3	2
T3–T4	2
T4–T5	2
T5–T6	1
T7–T8	4
T8–T9	2
T9–T10	2
T10–T11	4
T11–T12	8

### General Results

All tumors were completely resected in a single operation. The incidence of tumors in the lower thoracic segment (T8–T12) was 51.6% (16/31 cases), while the incidences in the upper thoracic segment (T1–T4) and middle segment (T5–T8) were 25.8% (8/31 cases) and 22.6% (7/31 cases), respectively. The mean intraoperative blood loss was 53.23 ± 33.08 mL (20–150 mL), and the mean operation time was 95.16 ± 20.31 min (60–180 min). There was no pleural or lung injury, and there were no complications, such as cerebrospinal fluid leakage. The average length of hospital stay was 5.75 (4–7) days.

### Clinical Improvement and Follow‐up

**Fig 4:**
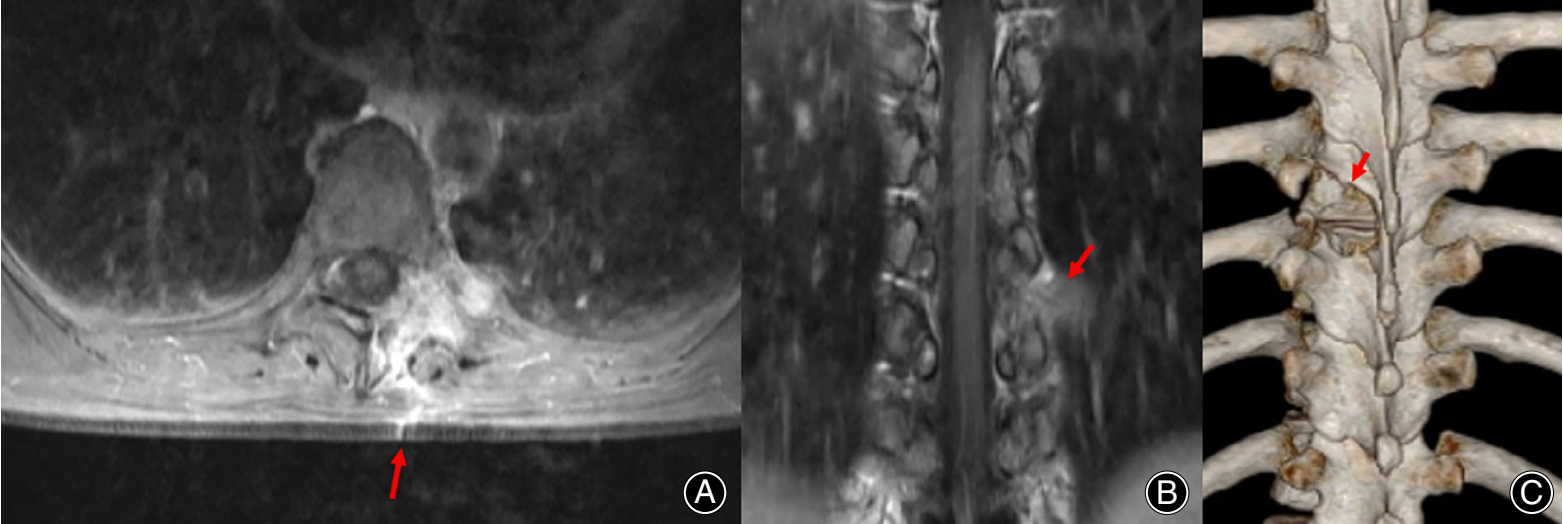
Postoperative T1‐weighted enhanced MRI on axial (A) and coronal (B) planes. Resection of the dumbbell tumor could not be identified (arrow) after 6 months. Postoperative 3D‐CT scan shows the resected vertebrae (C).

**Fig 5:**
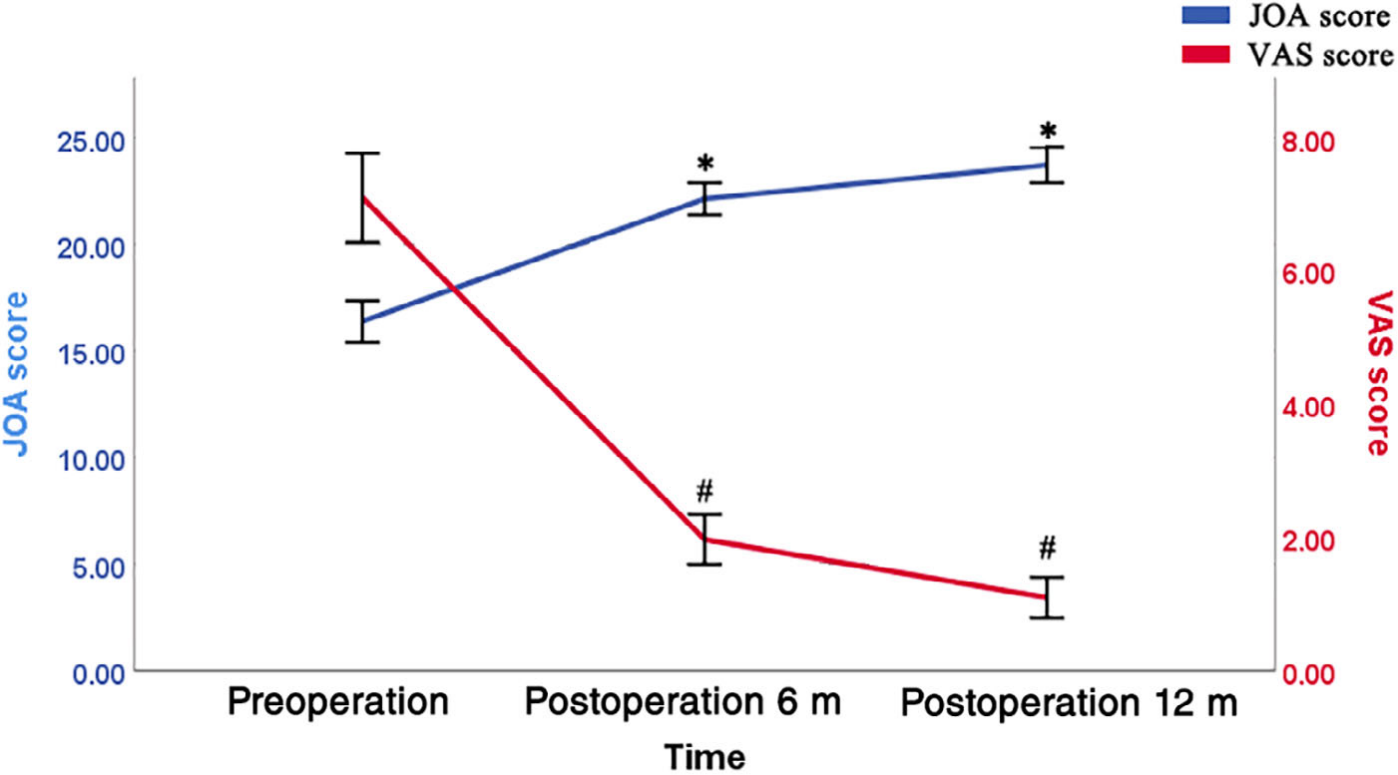
Preoperative and postoperative JOA and VAS score. Over time, the VAS score decreased gradually and the JOA score increased gradually. *P < 0.05 when comparing JOA scores with those of previous evaluation; #*P* < 0.05 when comparing VAS scores with those of previous evaluation. JOA, Japanese Orthopaedic Association score; m, month; op, operative; VAS, visual analogue scale.

Pain and lower limb weakness had been successfully treated in all patients at discharge. Over time, the VAS score decreased gradually and the JOA score increased gradually (Fig. 5). The VAS score significantly decreased and the JOA score significantly increased between the preoperative period and the postoperative follow‐up period (both *P* < 0.001) (Table 2). The average follow‐up time was 29 months (13–59 months), during which no tumor recurrence or spinal instability was observed. Typical cases are shown in Figs 1, 2, 3, 4.

**TABLE 2:** Surgical outcomes of the patients with dumbbell tumors (n = 31)

Variable	Measurement (mean ± SD)
Operation time	95.16 ± 20.31 min
Estimated blood loss	53.23 ± 33.08 mL
Complication	None
Postoperative hospital stay	5.29 ± 1.10 days
Follow up	29.26 ± 15.76 months
Preoperative VAS score*	7.09 ± 1.81
Postoperative VAS score*	0.48 ± 0.68
Preoperative JOA score**	16.39 ± 2.65
Postoperative JOA score**	25.39 ± 2.01

* Postoperative VAS score compared with preoperative VAS score, *P* < 0.001.

** Postoperative JOA score compared with preoperative JOA score, *P* < 0.001.

JOA, Japanese Orthopaedic Association; VAS, visual analogue scale.

### Subgroup Analysis

According to the subgroup analysis, there were significantly different JOA scores at 12 months postoperatively, operation times, and estimated blood loss among different types of tumors (*P* < 0.05) (Table 3). Furthermore, multiple comparison analysis of Fisher's least significant difference for different types of tumors was used and pairwise comparisons were performed. The group of Eden type II tumors had lower JOA scores at 12 months postoperatively, longer operation time, and more estimated blood loss compared with other groups (*P* < 0.05) (Table 4). There were no significant influences on VAS scores at 12 months postoperatively and postoperative hospital stay from the different types of tumors (Table 3).

**TABLE 3:** Subgroup analysis for Eden classification of spinal dumbbell tumors

Outcome	Mean ± standard deviation			*F*	*P‐*value
	Eden II	Eden III	Eden IV		
JOA					
Preoperative	14.25 ± 2.06	16.69 ± 3.17	16.72 ± 1.62	1.543	0.231
Postoperative 12 months	20.00 ± 1.83	24.63 ± 1.86	23.71 ± 2.30	10.657	0.000*
VAS					
Preoperative	7.50 ± 0.58	6.56 ± 2.28	7.73 ± 1.01	1.507	0.239
Postoperative 12 months	1.50 ± 0.58	1.19 ± 0.98	0.82 ± 0.60	1.201	0.316
Operation time (min)	127.50 ± 35.92	89.37 ± 9.98	91.82 ± 14.71	8.999	0.001*
Estimated blood loss (mL)	95.00 ± 42.03	54.69 ± 30.96	35.91 ± 16.86	6.413	0.005*
Postoperative hospital stay (days)	5.75 ± 0.96	5.31 ± 1.08	5.09 ± 1.22	0.515	0.603

* *P* < 0.05 suggests that there are differences between groups.

JOA, Japanese Orthopaedic Association; VAS, visual analogue scale.

**TABLE 4:** Multiple comparisons for Eden classification of spinal dumbbell tumors

Dependent variable	(I) Classification	(J) Classification	Mean deviation (I–J)	Standard error	Significance*	95% confidence interval	
						Lower limit value	Upper limit value
JOA postoperative 12 months	Eden II	Eden III	−4.62500	1.00185	0.000	−6.6772	−2.5728
		Eden IV	−3.72727	1.04640	0.001	−5.8707	−1.5838
	Eden III	Eden II	4.62500	1.00185	0.000	2.5728	6.6772
		Eden IV	0.89773	0.70195	0.211	−0.5401	2.3356
	Eden IV	Eden II	3.72727	1.04640	0.001	1.5838	5.8707
		Eden III	−0.89773	0.70195	0.211	−2.3356	0.5401
Operation time (min)	Eden II	Eden III	38.12500	9.16880	0.000	19.3436	56.9064
		Eden IV	35.68182	9.57649	0.001	16.0653	55.2984
	Eden III	Eden II	−38.12500	9.16880	0.000	−56.9064	−19.3436
		Eden IV	−2.44318	6.42411	0.707	−15.6024	10.7160
	Eden IV	Eden II	−35.68182	9.57649	0.001	−55.2984	−16.0653
		Eden III	2.44318	6.42411	0.707	−10.7160	15.6024
Estimated blood loss (mL)	Eden II	Eden III	40.31250	15.85169	0.017	7.8418	72.7832
		Eden IV	59.09091	16.55655	0.001	25.1763	93.0055
	Eden III	Eden II	−40.31250	15.85169	0.017	−72.7832	−7.8418
		Eden IV	18.77841	11.10647	0.102	−3.9722	41.5290
	Eden IV	Eden II	−59.09091	16.55655	0.001	−93.0055	−25.1763
		Eden III	−18.77841	11.10647	0.102	−41.5290	3.9722

* The significant level of mean difference is 0.05.

JOA, Japanese Orthopaedic Association; VAS, visual analogue scale.

## Discussion

### Selection of a Surgical Approach

Currently, to excise tumors in the thoracic cavity using the traditional posterior approach for thoracic dumbbell tumors, it is often necessary to make a large incision and remove part of the ribs, transverse processes, and vertebral pedicles, to increase the exposure of the posterolateral structures. This leads to the need for spinal internal fixation to maintain stability of the spine after tumor removal^2^, ^8^, ^9^, ^10^. Ando *et al*. reported 16 cases of thoracic dumbbell tumors treated with one‐stage posterior approach surgery, in which laminotomy combined with costal transverse process resection were performed, followed by fusion and internal fixation after tumor resection. Although this surgical approach avoided a combination of the anterior approach to remove tumors, the mean blood loss was approximately 540 mL, significant injury was caused, and spinal stability was affected, thus necessitating internal fixation^11^.

The development and introduction of video‐assisted thoracoscopic surgery (VATS) has significantly reduced tissue injury in the anterior approach, but it is still difficult to treat tumors in the intervertebral foramen and spinal canal^12^. In thoracic dumbbell tumors, the tumor in the thoracic cavity is usually large, and the tumor in the spinal canal causes neurological symptoms. Therefore, the anterior thoracic surgery approach is often combined with a posterior laminectomy or hemilaminectomy to excise the tumor in the spinal canal. In several previous cases of thoracic dumbbell tumors treated with combined surgery, we worked together with thoracic surgeons to perform posterior paravertebral surgery combined with VATS, which achieved en bloc resection of the tumor within and outside the spinal canal without the need for spinal internal fixation. However, combined surgery requires more anesthesia and intubation. It is also necessary to change the body position during surgery, which may increase the chances of lung, blood vessel, and nerve injury. In addition, operation times are longer, and the costs are higher. Furthermore, respiratory function recovers slowly after surgery and complications may occur, such as pulmonary inflammation, atelectasis, arrhythmia, and pain. VATS has some limitations, such as thoracoscopy being difficult in the upper thoracic segment and near the costophrenic angle and the risk of damaging the great vessels and the cervicothoracic sympathetic ganglia^13^, ^14^.

### Advantages of Tubular Spine Surgery for Thoracic Dumbbell Tumors

Appropriate surgical approaches are currently selected based on comprehensive judgment after considering the segment where the tumor is located, the tumor size, and whether the tumor is intradural or epidural, among other factors. Chen *et al*. reported on the surgical treatment of 20 cases of thoracic dumbbell tumors, in which a supraclavicular approach was selected for C7–T1 paravertebral tumors (Eden type IV), VATS or thoracotomy for paravertebral tumors in other segments (Eden type IV), and posterior laminotomy combined with VATS or thoracotomy for tumors invading the intervertebral foramen and spinal canal (Eden type II or III). They reported that the mean intraoperative blood loss was 360 mL, and the mean duration of using a drainage tube was 3 days^15^.

With the development of minimally invasive techniques and devices, new surgical approaches have been used to remove thoracic dumbbell tumors in one operation and reduce unnecessary iatrogenic injuries caused by combining approaches^16^, ^17^, ^18^. Tubular or expandable retractors in tubular spinal surgery can expose tumors by expanding muscles in a stepwise manner, which not only reduces muscle and ligament injuries but also addresses tumor tissue in the epidural space. We successfully resected schwannoma in the lumbar spinal canal in 56 cases through the paravertebral approach using the tubular technique. Compared with the semi‐laminar approach, it caused less blood loss and muscle injury, and postoperative spinal stability was adequate^19^. Most thoracic dumbbell tumors are schwannomas with clear boundaries, which makes minimally invasive surgery possible^20^. Zairi *et al*. reported successful one‐stage resection in five cases of thoracic dumbbell tumors (Eden type III) through the costotransverse approach using a tubule with a diameter of 2.4 cm. The mean blood loss was 230 mL (50–500 mL), and the mean operation time was 219 min^21^. We applied the keyhole technique in spinal tubular surgery. By changing the direction and depth of the micro‐tubule during surgery to expose the intraspinal and extraspinal tumor tissue, we successfully resected thoracic dumbbell tumors of various types (Eden type II to type IV) in 31 patients.

Key findings from the surgeries include: (i) for intraspinal and extraspinal tumors, the intraspinal tumor and tumor tissue in the intervertebral foramen should be removed first, followed by the paravertebral tumor tissue; (ii) most of the laminae and joints should be retained, damage to paravertebral muscles and ligaments should be avoided, the bony space of the intervertebral foramen should be used as much as possible for tumor resection, and if the costotransverse joint and the costal capital joint block the visual field of the tubule, part of the joints can be ground off to expose the tumor tissue in the paravertebral and intervertebral foraminal area; (iii) the blood supply vessels of the tumor are mostly distributed near the intervertebral foramen and should be preferentially blocked; (iv) when resecting paravertebral tumors, the pleura can be better protected by first performing intratumoral piecemeal resection and then dissociating and resecting the capsule; and (v) after resection of Eden type II tumors, tight suturing of the dura mater is the key measure to prevent cerebrospinal fluid leakage^22^, ^23^.

### The Double Location Method

When choosing a surgical procedure, the location and size of the tumor and whether the bone structure is destroyed are factors that should be considered^22^, ^23^. Although dumbbell‐shaped tumors in different segments from T1 to T12 can be removed through the paravertebral approach using a micro‐tubular technique, this procedure is not recommended for patients with severe scoliosis because abnormal spine and rib positions will limit the direction and depth of the operating tubule during surgery. During surgical resection of thoracic dumbbell tumors through the paravertebral approach using a micro‐tubular technique, we found that a micro‐tubule with a diameter of 14 mm could easily enter the intercostal space in the segment where the tumor was located while maintaining a distance from the costotransverse joint and the costal capital joint to further reach the lateral side of the vertebral body and lamina. However, this reduced damage to the muscles and zygapophysial joints, thus lowering the incidence of spinal instability.

Accurate tumor localization is a prerequisite for a successful minimally invasive surgery. During the operation, we adopted a double localization method to ensure accurate tumor localization and the paravertebral approach using a C‐arm X‐ray. First, the intervertebral foramen invaded by the tumor was used to determine the position of the skin incision. Second, the costotransverse joint, as the bony landmark, determined the depth and direction of the working tubule after cutting the skin. Therefore, the tumor could be directly exposed, and the intervertebral foramen could be found more safely using the double localization method; further tumors could be accessed and removed (Fig. 1).

### Complications

Assessing the locations of the pleura and tumor capsule is key to reducing the incidence of postoperative pulmonary complications. If the pleura is ruptured, this will lead to complications, such as pneumothorax and atelectasis, and a thoracic drainage tube will be needed; therefore, protecting the pleura is of great importance^24^, ^25^, ^26^. Intraoperatively, we found that the pleura moved significantly with the patient's breathing, but the tumor capsule could not be moved significantly. In the process of dissociating the tumor capsule, the capsule was first identified, and a gelatin sponge was used to push the capsule off, or a detacher was used to dissociate the capsule, and the pleura was pushed off. When the tumor was vascularized or had significant connective tissue adhesion, microscopic scissors were used for sharp dissection of the capsule to avoid pleural damage. For Eden type II tumors, the technique used in handling the spinal dural incision was key to avoiding complications, such as cerebrospinal fluid leakage. Because the pleura in the thoracic cavity cannot be completely reduced in a short period of time after resection of thoracic dumbbell tumors, respiratory movement will produce a negative pressure suction effect on the dura mater, causing cerebrospinal fluid leakage and pleural effusion^24^, ^27^. Therefore, we chose 6–0 or 7–0 nylon threads to suture the dura mater intermittently and tightly. The spinal canal in the intervertebral foraminal area could be closed with autologous tissue (fat or muscle) combined with biomedical fibrin glue to reduce the negative pressure suction caused by respiratory movement, and the drainage tube was left *in situ* to reduce pleural effusion^26^, ^27^.

### Limitations

This surgical approach has some limitations. If the tumor invades two or more segments, if the tumor in the thoracic cavity is very large, if the tumor is a space‐occupying vascular lesion, or if the tumor invades mediastinal structures, such as the esophagus and thoracic aorta, open surgery or combined surgery is recommended^28^.

Compared to previous combined surgeries and other microsurgeries, our technique can be used to remove thoracic dumbbell tumors in one operation, with minimal blood loss (100 mL). This procedure only requires one approach and a single incision and does not require combined surgery. It also minimizes the operation time, allows rapid recovery, and reduces complications. Furthermore, it causes little damage to tissues, muscles, joints, and bony structures; therefore, it has little influence on spinal stability and does not require spinal internal fixation.

### Conclusion

The paravertebral approach using a micro‐tubular technique (diameter, 14 mm; non‐expandable) is a safe and effective minimally invasive surgical procedure for the treatment of thoracic dumbbell tumors located between T1 and T12. This surgical approach can remove various types of thoracic dumbbell tumors (Eden types II to IV) in one operation, significantly reduce intraoperative blood loss and postoperative complications, shorten hospital stay, and reduce the incidence of spinal instability.
